# The effect of scanning pathways on trueness and precision in full-arch optical impression

**DOI:** 10.1186/s12903-023-03101-z

**Published:** 2023-06-14

**Authors:** Shota Kuroda, Mamoru Yotsuya, Toru Sato, Ryuichi Hisanaga, Syuntaro Nomoto, Hideshi Sekine

**Affiliations:** Department of Fixed Prosthodontics, Tokyo Dental College, 2-9-18 Kandamisakicho Chiyoda-Ku, Tokyo, 101-0061 Japan

**Keywords:** Optical impression, Intra oral scan, Scanning pathway, Trueness, Precision

## Abstract

**Background:**

In this study, we investigated the effects of differences in scanning pathways during optical impression on the trueness and precision of full-arch impressions.

**Methods:**

Reference data were obtained using a laboratory scanner. All optical impressions were measured across the dental arch using TRIOS® 3 in four different pathways. The reference and optical impression data were superimposed using the best-fit method. The criteria for superimposition were based on the starting side of the dental arch (partial arch best-fit method, PB) and based on the full arch (full arch best-fit method, FB). The data were compared between the left and right molars (starting and ending sides). The scan deviations for trueness (*n* = 5) and precision (*n* = 10) were obtained for each group by calculating the root mean square (RMS) of the deviation at each measurement point. Visual observations using superimposed color map images revealed variations in trueness.

**Results:**

There were no significant differences in scanning time or amount of scan data between the four scanning pathways. Trueness did not differ significantly among the four pathways with respect to the starting and ending sides, regardless of the superimposition criteria. Precision with PB was significantly different between scanning pathways A and B, and pathways B and C for the starting sides, and between scanning pathways A and B, and pathways A and D for the ending sides. In contrast, there was no significant difference between the starting and ending side in pathways for FB. Regarding PB, color map images showed a large error range in the direction toward the molar radius for the occlusal surface and cervical regions on the ending sides.

**Conclusion:**

Differences in the scanning pathways did not affect trueness, regardless of the superimposition criteria. On the other hand, differences in the scanning pathways affected the precision of the starting and ending sides with PB. Scanning pathways B and D were more precise on the starting and ending sides, respectively.

## Background

In recent years, advances in digital technology have led to the digitization of prosthetic devices in the dental industry, centered on computer-aided design (CAD)/computer-aided manufacturing (CAM) systems. The production process consists of four main steps. The first step is to obtain an impression of the abutment tooth and dental arch; the second step is to digitize the model; the third step is to design a prosthetic device using CAD software; and the fourth step is to fabricate the prosthetic device using CAM, which is a method to indirectly digitize information in the oral cavity [1, 2]. Optical impression technology, which uses intraoral scanners (IOS) to directly digitize information in the oral cavity, has recently attracted attention [3–5]. An IOS can quickly build a three-dimensional model by capturing still images or videos of hard and soft tissues. The optical impression method is characterized by a shortened chair time that is more comfortable for patients because of less gagging with limited vomiting reflexes and oral opening function, prevention of infection and reduction in the use of materials, elimination of the need for storage space owing to the digitalization of impressions, and easy communication with the technical laboratory personnel.

Many studies on optical impression methods applied in crown prosthesis have focused on the compatibility of single crowns or short-span bridges with approximately four units [6–8]. With single-tooth optical impressions, the precision reported for IOS is comparable to that of conventional methods using silicone impression materials [9]. For short-span scans and implant-based prosthetic treatments, the risk of error with digitization is reportedly low [10, 11]. In recent years, there has been an increase in the precision of full-arch procedures requiring long-span reconstruction. Such cases are common in the clinical setting. However, an increase in measurement errors has been reported with an increase in the length of the measured teeth [2, 12]. An invitro study showed that the full-arch optical impression method had the same precision as that of conventional impression methods [13], although others have concluded that its precision is lower than that of conventional methods [6, 14, 15].

Factors contributing to scanning data error with IOS include differences in the scanning approach [16–18], IOS image type (photo or video), presence or absence of powder [19, 20], size of the scanner head [21], hand abrasions during scanning, and patient movement. These factors influence the scanning pathway for the full-arch. Many IOS from various manufacturers have suggested full-arch scanning pathways. However, several aspects of its applicability remain unclear. Therefore, the effect of differences between scan pathways on the accuracy of full-arch and tooth-level measurements has not been sufficiently investigated.

In this study, we investigated the effects of differences in scanning pathways during optical impressions on the trueness and precision of full-arch impressions.

## Methods

### Reference model

This study considered teeth located in the lower jaw, and a mandibular model (D-18-500H (GUB) -MF: NISSIN, Kyoto, Japan) was used as the reference model. Reference data were acquired by scanning the reference model with a dental scanner (D2000: 3 Shape, Copenhagen, Denmark).

### Measurement method

#### IOS

In this study, TRIOS® 3 (3 Shape, Copenhagen, Denmark) was used as the IOS, with the confocal method as the scanning principle. Scanning was performed according to the manufacturer’s instructions without any powder. Prior to scanning, the scanner tip was calibrated and preheated as instructed by the manufacturer.

#### Scan environment

**Fig. 1:**
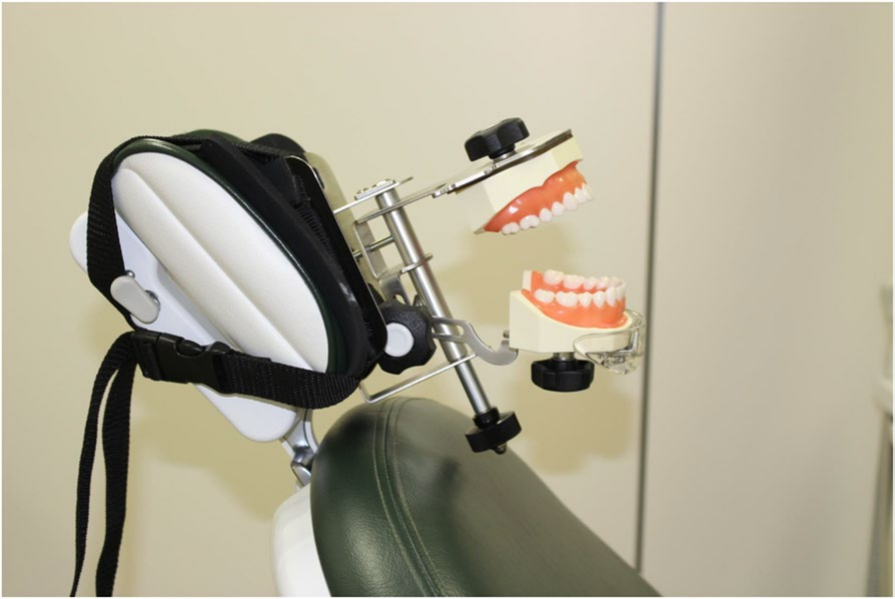
Scan environment. The reference model was set in a sitting position so that the occlusal plane was horizontal with the floor

The reference model assumed real clinical situations and mimicked patients placed in a dental unit (SIMPLE MANIKIN: NISSIN, Kyoto, Japan) (Fig. 1). The height of the unit was set to 50 cm from the floor so that the occlusal plane was horizontal and the back plate was inclined at 30°.

#### Scanning pathways and the time/amount of data

**Fig. 2:**
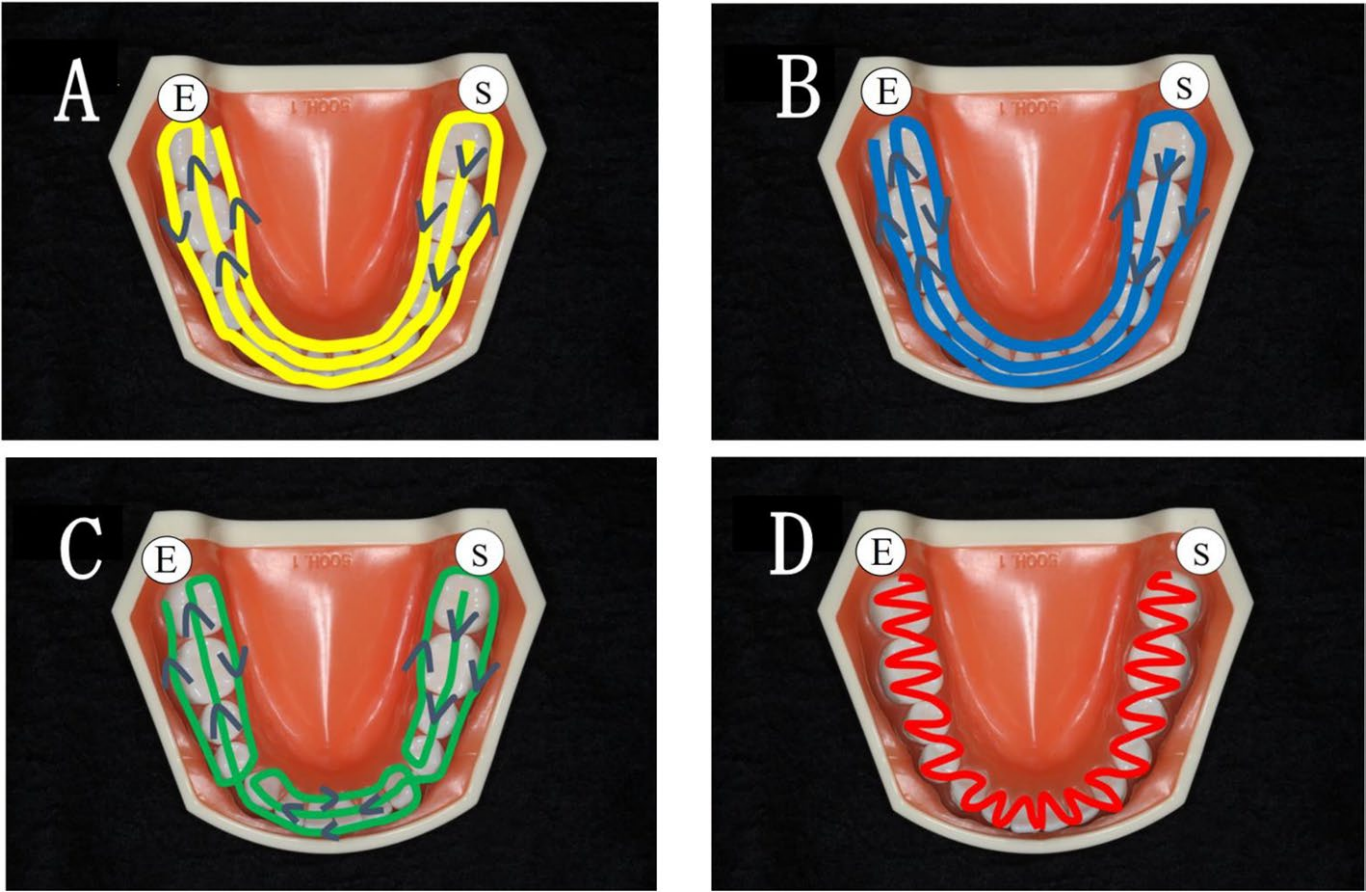
Four scanning pathways. Pathway **A**: Scan in the order of occlusal surface, buccal, and lingual sides. Pathway **B**: Scan in the order of occlusal surface, lingual side, and buccal side. Pathway **C**: Scan in a single sextant unit. Pathway **D**: Scan in an S-shape for each tooth. Ⓢ mark is the starting point (left second molar), and Ⓔ mark is the end point (right second molar)

Optical impressions involved the operator being positioned in front of the patient (8 o'clock position). The entire dental arch was measured five times each in four different scanning pathways (A, B, C, and D) from the left mandibular second molar (starting side) to the right mandibular second molar (ending side) (Fig. 2).

In pathway A, scanning was performed in the order of occlusal, buccal, and lingual surfaces. In pathway B, scanning was performed in the order of occlusal, lingual, and buccal surfaces. In pathway C, scanning was performed in the same order as that of pathway B for a single sextant up to the left mandibular first molar, and a series of scans were performed for a single sextant unit for the front and right mandibular molars. In pathway D, scanning was performed in an S-shape across all the teeth in single-tooth order on the lingual side, occlusal surface, and buccal side. The time required for scanning and amount of data acquired were recorded for each scan. The scans were performed sequentially without repeatedly stopping or resuming. All scans were performed by a dentist who was adequately experienced in scanning. After scanning, the images were confirmed to be unbroken and smooth digital images and were considered scans of acceptable quality for inclusion in the study.

#### Image analysis and data evaluation

All scan data were input into a CAD software (Dental System: 3 Shape, Copenhagen, Denmark) and converted to stereolithography (STL) data. Subsequently, the data were imported using 3D measurement software (Dmat3DE: DIGITAL PROCESS LTD., Kanagawa, Japan). After trimming excess soft tissue areas, such as gingiva, the data were superimposed using the best-fit method (vote-based pose estimation). Vote-based pose estimation is an algorithm that is used for 3D data superimposition [22]. In this method, the polygons of the entire dataset with minimal deviations are used for superimposition. The criteria for superimposition were set for two patterns, based on either the starting side of the dental arch (partial-arch best-fit method (PB)) or the full arch (full-arch best-fit method (FB)). The trueness of the starting- and ending-side molars was determined by superimposing the scan data of each scanning pathway with the reference scan data (*n* = 5).

The precision was superimposed between the scan data for each scanning pathway (*n* = 10). The precision was compared by combining two of the five measured data points. Therefore, the number of data points per scan pathway was 10.

Additionally, the color map image after superimposition for trueness was used to visually observe the deviation trend and determine the ± deviation between the two datasets. The deviation ranges of the starting and ending molars were visually observed, and the area of the greatest deviation in the color map of single-tooth units was selected as the representative value. Deviations included positive and negative values, with positive values shown as warm-colored, and reference data shown as convex (enlarged). In contrast, the negative values are cold-colored and the reference data are shown as pits (minimized). The minimum range of deviation (green area) was set to ± 50 µm and the maximum deviation value was set to ± 500 µm.

### Statistical analysis

Statistical analyses were performed using the Mann–Whitney U test and Kruskal–Wallis test to examine the effect of each scanning pathway on the trueness and precision of FB and PB with a significance level of 5%.

## Results

### Scanning time and amount of scan data

The results are shown in Table 1. The mean scanning time was 78.4 ± 4.5 s for scanning pathway A, 87.6 ± 4.3 s for scanning pathway B, 76.2 ± 1.0 s for scanning pathway C, and 83.2 ± 7.1 s for scanning pathway D.

**Table 1:** Average scanning time and data volume for each scanning pathway

	Pathway A	Pathway B	Pathway C	Pathway D
Average scanning time(Sec)	78.4 ± 4.5	87.6 ± 4.3	76.2 ± 1.0	83.2 ± 7.1
Average scanning data (Slices)	1065.2 ± 22.6	1168.2 ± 58.8	1055.2 ± 44.0	1108.8 ± 51.3

The average scanning time was less than 90 s, the average scanning data amount (slices) was approximately 1100 slices in all scan pathways, and there was no significant difference between the scan pathways.

The mean amount of scan data was 1,065.2 ± 22.6 for scanning pathway A, 1,168.2 ± 58.8 for scanning pathway B, 1,055.0 ± 44.0 for scanning pathway C, and 1,108.8 ± 51.3 for scanning pathway D.

### Scan deviation due to different scanning pathways

**Fig. 3:**
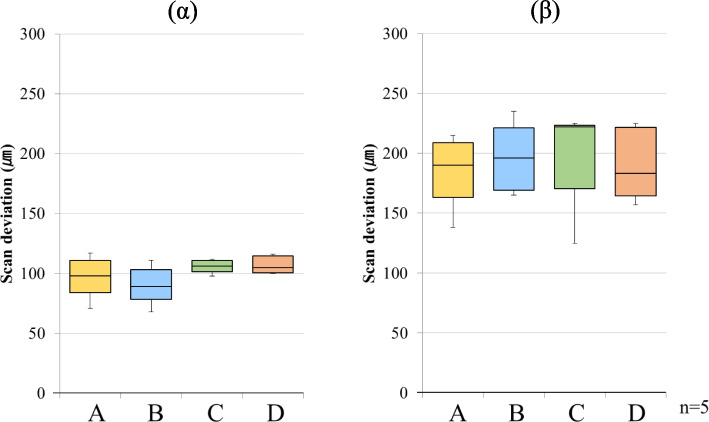
Scan deviation with PB for each scanning pathway. (α) starting side, (β) ending side. The box plots represent the scan deviation among data from the five test scans of the four scan pathways and the reference scan data (trueness)

**Fig. 4:**
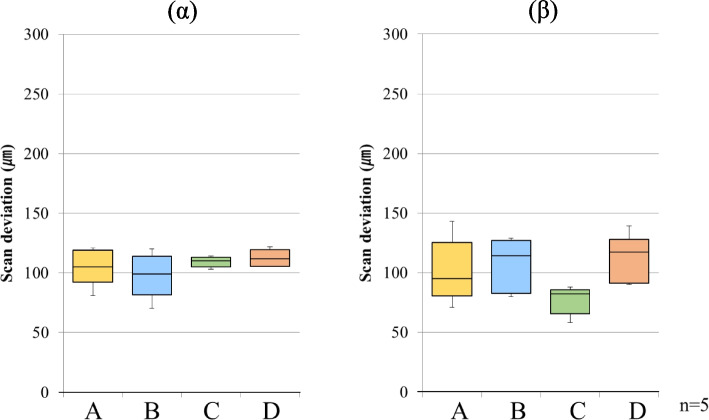
Scan deviation with FB for each scanning pathway. (α) starting side, (β) ending side. The box plots represent the scan deviation among data from the five test scans of the four scan pathways and the reference scan data (trueness)

**Fig. 5:**
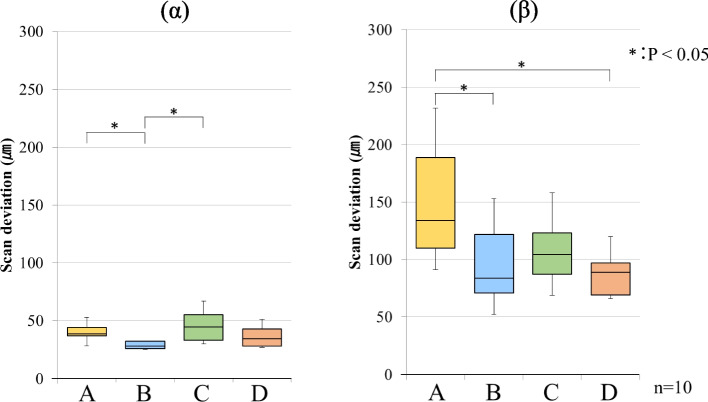
Scan deviation with PB for each scanning pathway. (α) starting side, (β) ending side. The box plots represent the scan deviation between each combination of data from the five test scans of the four scan pathways (precision)

**Fig. 6:**
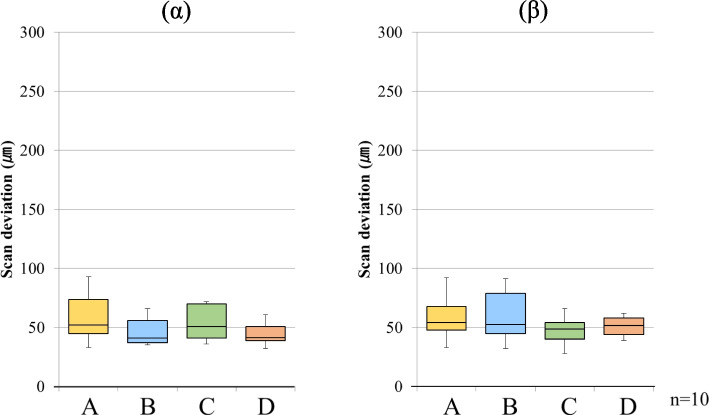
Scan deviation with FB for each scanning pathway. (α) starting side, (β) ending side. The box plots represent the scan deviation between each combination of data from the five test scans of the four scan pathways (precision)

The results of the scanning deviations for each group are shown as box plots in Figs. 3, 4, 5 and 6. The box plot contains 25 percentile and 75 percentile values of the scan deviation (median line, median), and the vertical lines represent the minimum and maximum values.

#### Trueness

For PB, the median starting side trueness was 98.0 µm (interquartile range = 26.0) for scanning pathway A, 89.0 µm (interquartile range = 24.5) for scanning pathway B, 106.0 µm (interquartile range = 9.5) for scanning pathway C, and 105.0 µm (interquartile range = 14.0) for scanning pathway D. The median ending side trueness was 190.0 µm (interquartile range = 46.0) for scanning pathway A, 196.0 µm (interquartile range = 52.0) for scanning pathway B, 222.0 µm (interquartile range = 53.0) for scanning pathway C, and 183.0 µm (interquartile range = 57.0) for scanning pathway D. The median starting side trueness was approximately 100 µm (interquartile range = 9.5–26.0) across all the scanning pathways. In contrast, the median ending- side trueness was approximately 200 µm (interquartile range = 46.0–57.0) across all scanning pathways, and the deviation was greater than that of the starting side trueness. In addition, there was no significant difference in trueness between the starting and ending sides for any scanning pathway (Fig. 3).

For FB, the median starting side trueness was 105.0 µm (interquartile range = 27.0) for scanning pathway A, 99.0 µm (interquartile range = 32.5) for scanning pathway B, 110.0 µm (interquartile range = 8.0) for scanning pathway C, and 112.0 µm (interquartile range = 14.0) for scanning pathway D. The median ending side trueness was 95.0 µm (interquartile range = 44.5) for scanning pathway A, 114.0 µm (interquartile range = 44.5) for scanning pathway B, 82.0 µm (interquartile range = 20.0) for scanning pathway C, and 117.0 µm (interquartile range = 37.0) for scanning pathway D. The median starting-side trueness was approximately 100 µm (interquartile range = 8.0–32.5) across all the scanning pathways. In contrast, the median ending side trueness was approximately 100 µm (interquartile range = 20.0–44.5) across all the scan pathways, and although the interquartile range was large, it was similar in size to the starting side trueness. In addition, there was no significant difference in trueness between the starting and ending sides for any of the scanning pathways (Fig. 4).

#### Precision

For PB, the starting side precision had median values of 38.5 µm (interquartile range = 7.0) for scanning pathway A, 28.0 µm (interquartile range = 6.0) for scanning pathway B, 44.5 µm (interquartile range = 22.0) for scanning pathway C, and 34.5 µm (interquartile range = 15.0) for scanning pathway D. Significant differences were observed between scanning pathways A and B, and between pathways B and C. For PB, the ending side precision had median values of 134.0 µm (interquartile range = 79.0) for scanning pathway A, 83.5 µm (interquartile range = 51.0) for scanning pathway B, 104.5 µm (interquartile range = 36.0) for scanning pathway C, and 89.0 µm (interquartile range = 28.0) for scanning pathway D. The median starting side precision was approximately 35 µm (interquartile range = 6.0–22.0) across all the scanning pathways. However, the median ending side precision was approximately 103 µm (interquartile range = 28.0–79.0) across all the scanning pathways. Significant differences were observed between scanning pathways A and B, and between pathways A and D (Fig. 5).

For FB, the starting side precision had a median value of 52.0 µm (interquartile range = 29.0) for scanning pathway A, 41.0 µm (interquartile range = 19.0) for scanning pathway B, 51.0 µm (interquartile range = 29.0) for scanning pathway C, and 41.5 µm (interquartile range = 12.0) for scanning pathway D. The ending side precision had a median value of 54.0 µm (interquartile range = 20.0) for scanning pathway A, 52.5 µm (interquartile range = 34.0) for scanning pathway B, 48.5 µm (interquartile range = 14.0) for scanning pathway C, and 51.5 µm (interquartile range = 14.0) for scanning pathway D. The median starting side precision was approximately 46 µm (interquartile range = 12.0–29.0) across all the scanning pathways. In contrast, the median ending side precision was approximately 50 µm (interquartile range = 14.0–34.0) across all the scanning pathways; although the interquartile range was large, it was similar in size to the starting side precision. In contrast to the PB, there was no significant difference between the starting and ending sides for any of the scanning pathways (Fig. 6).

### Color map images and deviation distribution

**Fig. 7:**
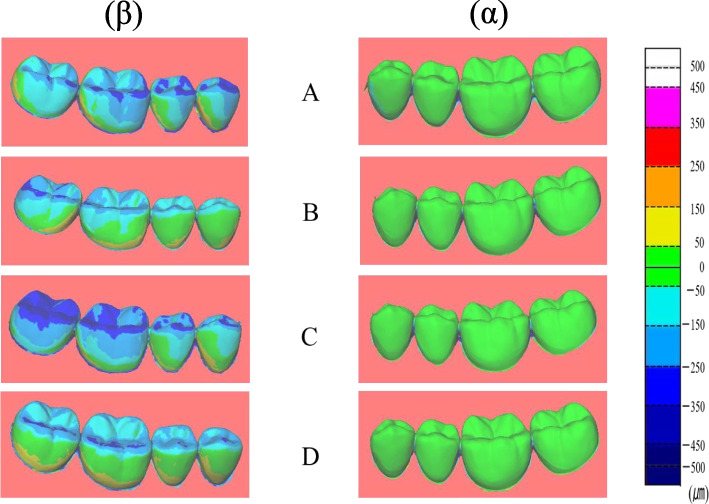
Representative example of color-coded deviation maps (trueness) between the reference and digital models for PB. (α) starting side: The deviation showed a range of ± 50 μm among all the scanning pathways, (β) ending side: The deviation range was -150 to -450 μm on the occlusal and buccal cervical regions

**Fig. 8:**
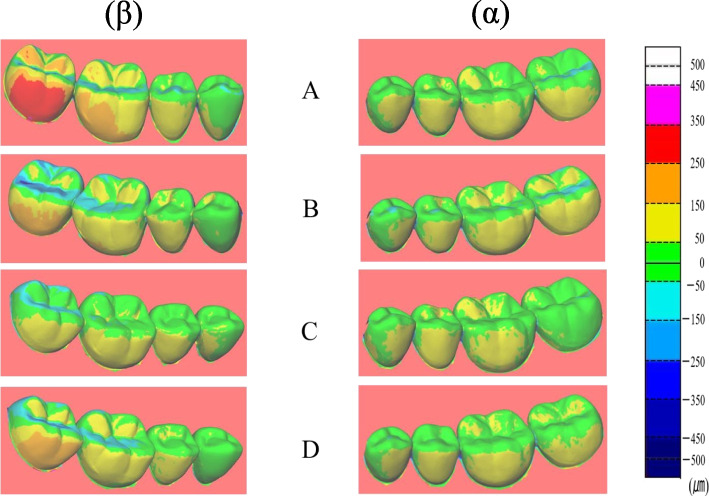
Representative examples of color-coded deviation maps (trueness) between the reference and digital models for FB. (α) starting side: The deviation mostly showed a range of + 50 to + 150 μm for all the scanning pathways, (β) ending side: The deviation showed a scattered range of ± 50 to ± 250 μm for all the scanning pathways

Figures 7 and 8 show the color maps generated by superimposing the trueness values. Tables 2, 3, 4 and 5 show the largest ranges of the color map trueness values using single-tooth units as representative values.

**Table 2:** Deviation distribution by tooth for PB starting side (trueness)

	A				**B**				**C**				**D**			
Type of tooth	34	35	36	37	34	35	36	37	34	35	36	37	34	35	36	37
(*µ*m)																
450~																
350 ~450																
250 ~350																
150~ 250																
50~ 150										**1**	**2**					
±50	**4**	**5**	**5**	**5**	**5**	**5**	**5**	**5**	**3**	**4**	**3**	**5**	**5**	**5**	**5**	**5**
-50~ -150	**1**								**2**							
-150~ -250																
-250~ -350																
-350~ -450																
-450~																

For all the color map images, the maximum deviation area was observed for each tooth

**Table 3:** Deviation distribution by tooth for the PB ending side (trueness)

	**A**				B				**C**				**D**			
Type of tooth	47	46	45	44	47	46	45	44	47	46	45	44	47	46	45	44
(*µ*m)																
450~																
350~450																
250~ 350																
150~ 250																
50~ 150																
±50																
-50~-150								**2**				**1**				
-150~ -250		**2**	**4**	**4**		**1**	**4**	**2**		**1**	**2**	**1**			**4**	**4**
-250~ -350	**1**	**1**	**1**	**1**	**1**	**2**	**1**	**1**	**1**	**2**	**3**	**3**	**1**	**4**	**1**	**1**
-350~ -450	**4**	**2**			**1**	**2**			**3**	**3**			**2**	**1**		
-450~					**3**				**1**				**2**			

For all the color map images, the maximum deviation area was observed for each tooth

**Table 4:** Deviation distribution by tooth for the FB starting side (trueness)

	A				B				C				D			
Type of tooth	34	35	36	37	34	35	36	37	34	35	36	37	34	35	36	37
(*µ*m)																
450 ~																
350 ~450																
250~ 350																
150~ 250																
50~ 150	**5**	**5**	**5**	**4**	**4**	**5**	**5**	**5**	**5**	**5**	**5**	**4**	**5**	**5**	**5**	**5**
±50				**1**	**1**							**1**				
-50~ -150																
-150~ -250																
-250~ -350																
-350~ -450																
-450~																

For all the color map images, the maximum deviation area was observed for each tooth

**Table 5:** Deviation distribution by tooth for the FB ending side (trueness)

	A				B				C				D			
Type of tooth	47	46	45	44	47	46	45	44	47	46	45	44	47	46	45	44
(*µ*m)																
450 ~																
350~ 450	**1**															
250~ 350					**2**								**1**			
150~ 250	**1**	**1**				**3**			**1**				**2**	**2**		
50~ 150	**3**	**4**	**4**	**4**	**1**	**2**	**5**	**5**	**2**	**5**	**5**	**5**	**1**	**3**	**5**	**5**
±50																
-50~ -150				**1**	**1**				**2**							
-150~ -250													**1**			
-250~ -350					**1**											
-350~ -450																
-450~																

For all the color map images, the maximum deviation area was observed for each tooth

With PB, the starting side trueness mostly showed a range of ± 50 µm for all the scanning pathways (Table 2). For the ending side, the deviation primarily ranged from -150 to -450 µm on the occlusal surface and buccal cervical regions (Table 3). This negative trend was particularly strong toward the molar distal region (Fig. 7).

With FB, the starting side trueness mostly showed a range of + 50 to + 150 µm for all the scanning pathways (Table 4). The ending side trueness showed a scattered range of ± 50 to ± 250 µm for all the scanning pathways (Fig. 8) (Table 5).

## Discussion

### Setting scanning pathway

In this study, we investigated the effect of scanning pathway on the trueness and precision of full-arch optical impressions, assuming long-span reconstruction. Past studies on optical impressions have been based on metal models made of Co-Cr alloys and Ti to minimize the deformation of focal dental models [1, 13, 23]. However, metallic reference models produce strong reflections on the surface of the models during scanning, which can result in a loss of data and scan failure. Therefore, an epoxy resin model with minimal reflection and high dimensional stability was used as the reference model [24]. Optical impressions were obtained using reference models attached to mannequins placed in the dental unit to mimic standard patient conditions [25]. To suppress the reflection of the model surface as much as possible during the scan, non-shadow lamps were avoided in favor of natural lighting according to the manufacturer’s instructions.

In this study, four pathways were chosen to scan the optical impressions. In all the pathways, scanning was performed from the left mandibular second molar occlusal surface [26] to facilitate stitching during scanning. Scanning pathways A and B were initially common only to the occlusal surface, but we also scanned the opposite molar regions. When using optical impression for prosthetics, it is recommended that the scanning range should not exceed a single sextant [2, 27, 28]. Accordingly, pathway C was scanned as a single sextant unit. Finally, pathway D was scanned in single-tooth units, either in an S-shaped or zigzag pattern [23, 24], primarily to allow for effective scanning of the anterior teeth. We did not perform a single broad scan for pathways C or D. Instead, we scanned a single sextant or tooth-by-tooth. Thus, the four scanning pathways can be separated into two large scanning ranges that operate simultaneously. Therefore, this study allowed us to consider how differences in the scanning range (distance) affect the trueness and precision of three-dimensional data.

### Best-fit method

Post-scan three-dimensional data were superimposed on reference data or acquired data using three-dimensional measurement software after conversion to STL data. The D2000® dental laboratory scanner (3 Shape, Copenhagen, Denmark) used to scan the reference model adopted a multiline scanning method using blue light-emitting diode (LED) light with a scanning precision of ± 5 µm. Unlike IOSs, dental laboratory scanners can measure a wide range of angles using high-performance cameras while shielding from natural light. Thus, this technology allows us to obtain high-precision data and has been used in several studies to obtain reference data [12].

Several studies have reported the trueness and precision of optical impression superimposition, citing three-dimensional data obtained using best-fit methods [2, 4, 15, 23, 28–31]. The characteristics of this method include visual representation of the entire three-dimensional model depression, making it possible to assess displacement by color mapping [1, 10, 14, 32].

However, this method is not suitable for evaluating errors at particular points (such as the distances between the centers of multiple ball abutments). Therefore, a best-fit algorithm has not been used in implantology research. Rather, these studies were evaluated by determining the central coordinates of two ball abutments and calculating the distance between them [12, 29]. A disadvantage of this method is that the variation within the entire model cannot be evaluated stereoscopically because the error is evaluated as the distance between the central coordinates at arbitrary points. This method may also lack reproducibility and incorporate measurement bias [29, 31]. In this study, data superimpositions were performed using the aforementioned best-fit algorithm, which allowed the assessment of dentition for studying optical impression methods in a fixed prosthodontic region.

### Selecting best-fit method criteria

Most studies using the best-fit algorithm overlapped with the full arch using the least-squares method [14, 33]. When using the least squares method for superimposition, the software adjusts the parameters such that there are no significant deviations or variations in the data as a whole. Therefore, this method is suitable for assessing whether the target and reference objects are similar in shape. In this study, we used vote-based pose estimation, which is a best-fit algorithm. In this method, polygons of the entire dataset with minimal errors were used for superimposition. In this case, polygons with large errors were not used for superimposition, resulting in small areas with small deviations and large areas with large deviations.

Therefore, this method is suitable for measuring the deviations between datasets. The algorithm most likely attempted to register the surfaces such that the overall mean deviation between the surfaces was minimized, which may conceal an increase in deviation between the surfaces and make the interpretation of the deviation difficult. A best-fit algorithm based only on areas where the scan started may show an increase in deviation [16]. In this study, a scan with the left molar region as the starting side and a full-arch scan were performed to create two reference superimpositions.

#### Effects on scanning time and data amount

The scanning time was shorter than that of conventional methods using silicone impression materials for all the scanning pathways [25]. In addition, the amount of scanned data did not exceed the upper limit (2000 slices) for optimal post-scan data transmission, and the scan path settings used in this study were considered appropriate for standard clinical applications.

#### Effects on scanning pathway trueness

The IOS accuracy was assessed primarily using two measures: “trueness” and “precision” (ISO 5725–1) [34]. Trueness is defined as the deviation from reference data values [13, 34–36].

There was no significant difference between the starting and ending sides for either PB or FB for any of the scanning pathways. Depending on the digital system, there have been reports [30] that show no effect of the different scanning pathways on trueness. Another report [24] suggests that the trueness and precision of TRIOS®, Omnicam®, and 3 M™ True Definition Scanner are unaffected by the differences in scanning method when recording impressions over long spans. These results are consistent with the findings of this study. Therefore, in this study, the degree of authenticity was not affected by differences in scanning pathways. Many previous studies used the second-generation TRIOS® Pod or TRIOS® Color [2–6, 9, 12]. In this study, the third-generation TRIOS® 3 was used. Although there were differences in scan speed and system version, TRIOS® 3 had the common scanning principle and was powderless.

PB and FB trueness showed similar values for the starting side; however, for PB, the trueness of the ending side was lower than that of FB.

#### Effects on scanning pathway precision

Precision may refer to reproducibility and is defined as a measure of how close similar values are to each other, independent of the reference data [13, 34–36]. There was no significant difference in precision between the starting and ending sides of PB for any scanning pathway. In addition, there was no significant difference in precision between the starting sides and ending sides of FB for any scanning pathway.

The scan deviation at the starting side of PB showed the highest precision with scanning pathway B. Significant differences were observed in precision between scanning pathways A and B, and between pathways B and C. Müller et al. investigated the effect of three scanning pathways on optical impression trueness and precision using Trios® Scanner with a maxillary full-arch model [23]. In their study, after scanning the occlusal and palatal sides, the scan pathway on the buccal side showed the highest precision, which is consistent with the results of our study. Scanning pathways in the order of the occlusal surface, palatal side, and buccal side have been reported to eliminate the risk of increasing errors when using linear data acquisition over a longer distance. Therefore, regarding the precision of the starting side, it is believed that scanning pathway B displays similarly high precision as scanning pathway A, which first scans a wide range. The scan deviation of the ending side of PB showed the highest precision for scanning pathway D. Significant differences were observed in precision between scanning pathways A and B, and between pathways A and D. Factors influencing deviation among the molars of the ending side included increased scan distance and scanning of the anterior teeth. Large deviations at the radial end of the scan data have been reported because of the accumulation of overlapping deviations in the anterior dental region [10]. The fact that anterior teeth are structurally simple makes accurate stitching of data particularly difficult [6, 14]. It was suggested that reducing the scan range, such as for scanning pathways C and D, may reduce the deviation in the anterior region compared with a wider scan. Finally, pathway D, which involved wide scanning, showed higher precision than pathway A.

For FB, the precision on the starting side was approximately the same as that on the ending side, and no significant difference was observed.

In addition, similar precision was observed for PB and FB on the starting side, as was the case with trueness. However, in the case of PB, the precision on the ending side was lower than that of FB.

### Trueness color map image deviation trends

Superimposed trueness data are displayed in a color map format. As the starting side was considered as standard for the superimposition of PB trueness, the starting side trueness frequently showed a range of ± 50 µm for all the scanning pathways. For the ending side, the deviation mainly ranged from -150 to -450 µm on the occlusal surface and buccal cervical regions.

Previous studies have reported that TRIOS® shows the largest deviation in the molar region and tends to marginally underestimate the reference file [35]. This finding is consistent with the results of the present study.

The trueness of FB was in the range of + 50 to + 150 µm, with the majority of the deviation on the starting side. On the ending side, the deviation ranged from ± 50 to ± 250 µm. In addition, the degree of deviation was strong enough to tend toward the molar distal regions. Many studies using superimposed full-arch color map images have reported large displacements in both the vertical and horizontal directions in the radial molar region of the ending side [4, 10, 14, 23, 29, 37]. It has also been reported that deviation from the occlusal surface increases owing to the strong effect of factors such as image overlap and still image skill [26]. Similarly, in this study, we believe that the deviation range of the ending side showed a strong tendency toward vertical or horizontal displacement of the occlusal surface and the cervical region, tending toward the distal region.

### Clinical significance

In this study, the trueness and precision of the full arch could be evaluated on the ending side when the two datasets were superimposed. On the ending side, the trueness of PB was 183.0 ~ 222.0 µm, and that of FB was 82.0 ~ 117.0 µm. The precision of PB was 83.5 ~ 134.0 µm, and that of FB was 48.5 ~ 54.0 µm. The accuracy of optical impressions for full-arch teeth has been reported to be within an acceptable range of 250 µm or less [26]. Thus, it was suggested that there is no issue with the reproducibility for jaws with teeth.

In contrast, the trueness and precision of the partial arch can be evaluated on the starting side, where the two datasets are superimposed. For the starting side, the trueness of PB was 89.0 ~ 106.0 µm and that of FB was 99.0 ~ 112.0 µm. The precision of PB was 28.0 ~ 44.5 µm, and that of FB was 41.0 ~ 52.0 µm, indicating a higher precision than that for full arch. This suggests that the partial-arch prosthesis range is more accurate than the full-arch range. If the final prosthetic device is within the range of one sextant, it is ideal to perform a scan.

Visual observation of color map images for trueness indicated that the starting side trueness for PB ranged between ± 50 µm and the starting side trueness for FB ranged from + 50 to + 150 µm. From these results, it can be inferred that in the case of FB, the abutment teeth were expressed to a slightly greater extent.

In contrast, the trueness of ending side of PB ranged from -150 to -450 µm, and the trueness of ending side of FB ranged from ± 50 to 250 µm. From these results, it can be inferred that a one-sided prosthesis with better trueness may be superimposed by PB. Furthermore, FB is preferred if a bilateral prosthesis is present. Because the color map was visually observed from only one direction (the buccal side), the actual displacement of the lingual-side data was unclear. Therefore, in future studies it is necessary to observe from other directions.

In addition, the scan deviation results and color map image observations did not necessarily match. The scan deviations are expressed as absolute deviations. However, the color map image is in single-tooth units, with the largest color map trueness range as a representative value. Therefore, we need to evaluate the results of both scanning pathways for trueness impact.

In this study, the abutment teeth were not prepared in the model because natural dentition was assumed. It has been reported that the shape of the prepared abutment teeth surface may affect the accuracy of the optical impression [33], and that scanning of the mucosal and subgingival areas is difficult [3, 21]. It is important to investigate the shape and margin of the abutment teeth and mucosal deficiencies.

A limitation of this study was that all scans were performed by a single dentist. Although the dentist was well-trained before scanning the models, operator bias during scanning cannot be excluded. Five scans were performed for each scan pathway, which may not be statistically significant in case of relatively large differences between the compared groups. This needs to be tested because increasing the number of scans may increase the detection power. Furthermore, this study was conducted in vitro, and factors that could affect the scans, such as saliva or moving soft tissues present in the actual oral cavity, were absent. Therefore, these factors were not considered in this study. In a study analyzing the in vivo trueness and precision of the maxillary dental arch, the authors reported that when the entire arch was superimposed, there was no specific error pattern in color-mapped images with TRIOS® 3 precision, and the errors could not be localized to a specific area or side of the dental arch [38]. Therefore, clinical conditions may show error patterns different from those obtained in this experiment, and the effects of different scan pathways need to be tested in vivo.

## Conclusions

In this study, two superimposition criteria for PB and FB were used for 3D optical impression data using four scan pathways. The trueness and precision of the starting and ending sides of the molar were evaluated. The experiments produced the following results:

1. The scans using the four scan pathways in this study were within the clinically acceptable range in terms of scanning time and amount of scan data.
2. The scanning pathways did not affect the trueness of the starting and ending sides both for PB and FB.
3. The scanning pathways affected the precision of the starting and ending sides for PB. Scanning pathways B and D were more precise on the starting and ending sides, respectively. For FB, the scanning pathway did not affect the precision of both the starting sides and ending sides.

## Data Availability

Data are available from the corresponding author after approval by all authors.

## Abbreviations

PB: Partial-arch best-fit method
FB: Full-arch best fit method
RMS: Root mean square
CAD: Computer-aided design
CAM: Computer-aided manufacturing
IOS: Intraoral scanners
STL: Stereolithography
